# Incidence of acute lymphocytic leukemia in Calgary, Alberta, Canada: a retrospective cohort study

**DOI:** 10.1186/s13104-018-3225-9

**Published:** 2018-02-07

**Authors:** Rayven Snodgrass, Leonard T. Nguyen, Maggie Guo, Marcus Vaska, Christopher Naugler, Fariborz Rashid-Kolvear

**Affiliations:** 1 Department of Pathology and Laboratory Medicine, Cumming School of Medicine, University of Calgary, Calgary, AB Canada; 2 Calgary Laboratory Services, Calgary, AB Canada; 3 Knowledge Resource Service, Alberta Health Services, Calgary, AB Canada; 4 Department of Family Medicine, Cumming School of Medicine, University of Calgary, Calgary, AB Canada; 5 Cancer Cytogenetics Laboratory Centre, Calgary, AB Canada; 6 Department of Medical Genetics, Cumming School of Medicine, University of Calgary, Calgary, AB Canada; 7 C262, Diagnostic and Scientific Centre, 9 3535 Research Road NW, Calgary, AB T2L2K8 Canada

**Keywords:** Acute lymphoblastic leukemia, Childhood leukemia, Cancer epidemiology, Hematologic neoplasm, Canada

## Abstract

**Objective:**

Acute lymphocytic leukemia (ALL) is a rare malignant neoplasm that develops from abnormal lymphoid stem cells. ALL incidence is highest among children and declines towards adolescence. There is limited data on the epidemiology of ALL, especially in Canada. This retrospective cohort study used patient data from the Calgary Laboratory Services Cancer Cytogenetics Laboratory to report the incidence rate of ALL in Calgary, Alberta, Canada. New cases of ALL were identified for the 5-year period of January 1, 2011 until December 31, 2015. Reported incidence rates were categorized by sex and age groups, and age-standardized to the Canadian population.

**Results:**

There were an average of 11.4 new cases of ALL diagnosed per year between 2011 and 2015. The total incidence rate per 100,000 person-years was 0.84. Incidence rates peaked in children aged 0–4 with 7.55 and 3.32 cases per 100,000 person-years for males and females, respectively. The median age of diagnosis was 8 years. Incidence rates were generally lowest for adults aged 20 and over. The ratio of males to females diagnosed with ALL was 1.59. Overall, the recent incidence of ALL in Calgary is comparatively low with a preference for males and children below 5 years of age.

## Introduction

Acute lymphocytic leukemia (ALL) is a rare malignant disorder that develops from abnormal lymphoid stem cells and results from the clonal proliferation of lymphoid precursors with arrested maturation [1]. The disease can originate in lymphoid cells that give rise to B cells, T cells or sometimes both in the case of mixed-lineage leukemia [2]. T cell ALL occurs in approximately 15% of cases while B-cell ALL appears in up to 85% of cases [3]. ALL is of particular interest because it is predominantly a childhood disease (ages 0–14) [4], accounting for approximately 25% of cancers and 80% of all leukemias in children [5]. The incidence of ALL peaks sharply among children 1–4 years of age, accounting for 50% of ALL patients, with a slow decline towards adolescence [6–8]. ALL comprises < 1% of adult cancers and approximately 20% of adult leukemias [2, 5].

Between 2008 and 2012, a total of 532 childhood cancer cases were diagnosed in Alberta [4]. During this time period, the most commonly diagnosed childhood cancers were leukemia (27%), central nervous system cancers (23%), and lymphoma (10%) [4]. Of the childhood leukemia cases, 79% were ALL and 16% were acute myeloid leukemia [4]. Although the prognosis for ALL is particularly poor among adults, approximately 90% of childhood cases are cured [9]. This dramatic improvement in survival outcome has occurred over the last 2–3 decades and has been attributed to combination chemotherapy and cooperative clinical trials [10]. These therapeutic advances in childhood cancer research have relied on well designed and accurate epidemiological studies.

In general, the age-standardized incidence of ALL is highest in the Americas and Oceania and lowest in Asia and eastern Europe [5]. Italy, the United States (US), Switzerland, and Costa Rica are the countries with the highest incidence of ALL [11]. The lowest rates of ALL are found in developing countries, however concerns are frequently raised related to statistics produced in these countries [12]. Potential reasons include misdiagnosis, underreporting or pre-emptive death from infectious disease. Although not clearly understood, differences in incidence rates between ethnic groups may be related to hereditary link, genetic defects, and environmental risk factors such as radiation or chemical exposure. It is also noted that the incidence of ALL is slightly higher in males compared to females and in Caucasians compared to people of African descent [11].

Despite dramatic improvement in the understanding of the pathophysiology of ALL and survival outcomes for children [9], there is still limited data on the epidemiology of the disease. In particular, there are a limited number of studies on the incidence of ALL in Canada. Furthermore, statistical studies on ALL are typically limited to children and do not include data on older adults. The purpose of this study was to provide age and sex-categorized data on the incidence of ALL in Calgary, Alberta, Canada between 2011 and 2015.

## Main text

### Methods

#### Ethics statement

Ethics approval for this study was obtained from the Health Research Ethics Board of Alberta Cancer Committee (Ethics ID HREBA.CC-16-0830).

#### Source of data

This study was designed as a retrospective cohort. Patient data was obtained from the Calgary Laboratory Services (CLS) Cancer Cytogenetics Laboratory. The facility receives all new bone marrow samples from its catchment area which includes over 1.8 million residents in Calgary and the surrounding southern Alberta area. The samples are examined by flow cytometry and microscopic hematopathology analysis. The resulting diagnostic reports are evaluated by a cancer cytogeneticist to order the appropriate cytogenetic tests for further analysis. Incident cases of ALL were identified for the 5-year period of January 1, 2011 until December 31, 2015. Patient postal codes were used to exclude patients living outside the City of Calgary.

#### Incidence rate calculations

New ALL cases were categorized by sex and 5-year age groups and incidence rates with 95% confidence intervals were calculated using published methods [13]. Calgary population data used in the calculations was taken from Statistics Canada’s CANSIM database in the form of revised and updated population estimates determined by postcensal coverage studies [14]. These estimates provide more accurate measures of population counts than the 2011 census questionnaire, in part by taking into account residents who were missed by the census. The incidence rate was then standardized to the Canadian population by age groups using estimates for each year from CANSIM [15].

### Results

**Fig. 1:**
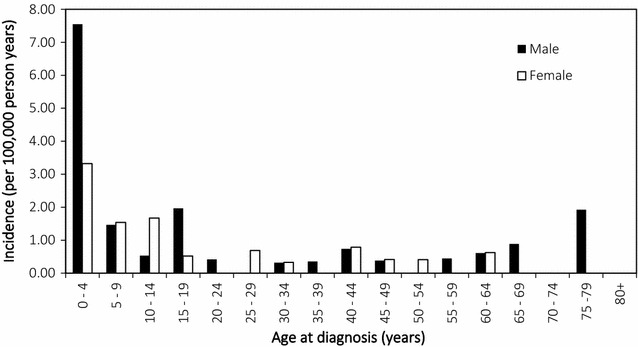
Age and sex-categorized incidence rates of acute lymphoblastic leukemia in the Calgary metropolitan area (2011–2015)

There were an average of 11.4 cases of ALL diagnosed per year in the Calgary metropolitan area between 2011 and 2015 (Table 1). The total incidence rate was 0.84 cases per 100,000 person-years, and the age-standardized rate for Canada is 0.79 cases per 100,000 person-years. The median age of diagnosis was 8 years. Slightly more males were diagnosed with ALL compared to females with incidence rates 1.02 and 0.66 cases per 100,000 person-years, respectively. Figure 1 shows age- and sex-categorized incidence rates of ALL in this population. Incidence rates peaked in children aged 0–4 with 7.55 and 3.32 cases per 100,000 person-years for males and females, respectively. Incidence declines sharply for adults aged 20 and over. There was a spike in incidence for 75–79 year old males, reflecting one new case within the 5 year period of study.

**Table 1:** Incidence features of acute lymphoblastic leukemia in the Calgary metropolitan area (2011–2015)

Total new cases (per year)	11.4
Total incidence rate (per 100,000 person-years)	0.84
95% confidence intervals	(0.64–1.1)
Canadian incidence rate (age-standardized, per 100,000 person-years)	0.79
95% confidence intervals	(0.74–0.83)
Male/female	1.59
Median age of diagnosis (years)	8
Age range (years)	0–78

### Discussion

In this study, we determined the incidence of childhood and adult ALL in Calgary, a major Canadian city, for the study period of 2011–2015. This has been similarly performed by our group for acute myeloid leukemia [16]. Patient data was obtained from the CLS Cancer Cytogenetics Laboratory in Calgary, Alberta. Our study provides important incidence rates of ALL over a recent 5-year period separated by age groups and sex.

The total incidence rate was 0.84 cases per 100,000 person-years with an average of only 11.4 new cases diagnosed per year in the metropolitan area with an average 1.3M population. This is relatively low compared to previous studies which report age-standardized incidence estimates of ALL ranging from 1 to 4 cases per 100,000 depending on geographical location [5, 17]. The age-standardized rate of ALL for Canada is 0.79 cases per 100,000 person-years.

ALL incidence varies considerably by age, with peak incidence rates for children aged 0–4 and over 50% of cases occurring in children 10 years old or under. This agrees with the literature and in most countries, the incidence rate of ALL in children is approximately three to four times that in adults [5, 10, 17]. Lower incidence rates were observed in adults past the age of 20, however there are some age categories with slightly increased incidence, i.e. 40–44 and 75–79 year olds. ALL was absent in individuals aged 80 years and older. We also report a higher ratio of males to females with ALL (1.59), which is comparable to other epidemiology studies (1.24–1.70) [17–19].

Although a rare disease, ALL presents a significant public health burden given poor survival outcomes among adults [9]. ALL is also of importance because it is one of the most common pediatric cancers [5]. Thus, quantification of the projected number of annual ALL diagnoses are central to understanding the societal burden of this disease. Future studies are warranted to monitor epidemiological trends such as incidence of ALL in cities of similar size to Calgary. These epidemiology studies should explore additional characteristics of the population of interest including survival/mortality rates, socioeconomic status and potential causal agents.

In conclusion, we report a relatively low incidence rate of 0.84 new cases per 100,000 for ALL in the Calgary Metropolitan Area, with over 50% of cases occurring in children under 10 years old.

## Limitations

While our study covered several years of new data, we acknowledge some limitations. The data gathered for this particular study is based on one geographical region at the time of diagnosis. In addition, our study looks strictly at the epidemiology of ALL and thus excludes data on potential causes, such as toxins and other infectious or causative agents. Lastly, the study does not include relevant data on the survival rate and sociodemographic correlates of individuals diagnosed with ALL.

## Abbreviations

ALL: acute lymphocytic leukemia
CLS: Calgary Laboratory Services
